# Genetic and metabolic comparison of orthotopic and heterotopic patient-derived pancreatic-cancer xenografts to the original patient tumors

**DOI:** 10.18632/oncotarget.23567

**Published:** 2017-12-21

**Authors:** Eunsung Jun, Seung-Mo Hong, Hyun Ju Yoo, Moon-Bo Kim, Ji Sun Won, Soyeon An, In Kyong Shim, Suhwan Chang, Robert M. Hoffman, Song Cheol Kim

**Affiliations:** ^1^ Division of Hepato-Biliary and Pancreatic Surgery, Department of Surgery, University of Ulsan College of Medicine and Asan Medical Center, Seoul, South Korea; ^2^ Department of Biomedical Sciences, University of Ulsan College of Medicine, Seoul, South Korea; ^3^ Department of Pathology, University of Ulsan College of Medicine and Asan Medical Center, Seoul, South Korea; ^4^ Department of Convergence Medicine, Asan Institute for Life Sciences, University of Ulsan College of Medicine and Asan Medical Center, Seoul, South Korea; ^5^ MetaBio Inc., Gangdong-Gu, Seoul, South Korea; ^6^ Asan Institute for Life Science, University of Ulsan College of Medicine and Asan Medical Center, Seoul, South Korea; ^7^ Department of Surgery, University of California, San Diego, CA, USA; ^8^ AntiCancer Inc., San Diego, CA, USA

**Keywords:** pancreatic cancer, patient-derived orthotopic xenograft PDOX, patient-derived heterotopic xenograft PDHX, homology, heterogeneity

## Abstract

Tumors from 25 patients with pancreatic cancer were used to establish two patient-derived xenograft (PDX) models: orthotopic PDX (PDOX) and heterotopic (subcutaneous) PDX (PDHX). We compared gene expression by immunohistochemistry, single-nucleotide polymorphism (SNP), DNA methylation, and metabolite levels. The 4 cases, of the total of 13 in which simultaneous PDHX & PDOX models were established, were randomly selected. The molecular-genetic characteristics of the patient's tumor were well maintained in the two PDX models. SNP analysis demonstrated that both groups were more than 90% identical to the original patient's tumor, and there was little difference between the two models. DNA methylation of most genes was similar among the two models and the original patients tumor, but some gene sets were hypermethylated the in PDOX model and hypomethylated in the PDHX model. Most of the metabolites had a similar pattern to those of the original patient tumor in both PDX tumor models, but some metabolites were more prominent in the PDOX and PDHX models. This is the first simultaneous molecular-genetic and metabolite comparison of patient tumors and their tumors established in PDOX and PDHX models. The results indicate high fidelity of these critical properties of the patient tumors in the two models.

## INTRODUCTION

Patient-derived xenografts (PDX) models of cancer have demonstrated important utility for the study of the mechanism of cancer, novel drug discovery and evaluation and personalized, precision medicine [1, 2].

There are two major types of PDX models: the orthotopic model, termed patient-derived orthotopic xenograft (PDOX) and a heterotopic model with subcutaneous implantation termed patient-derived heterotopic xenograft (PDHX).

Toward the goal of precision, personalized oncology, our laboratory pioneered the patient-derived orthotopic xenograft (PDOX) nude mouse model with the technique of surgical orthotopic implantation (SOI), including pancreatic [3–8], breast [8], ovarian [9], lung [10], cervical [11, 12], colon [13–15], stomach [16], sarcoma [17–28] and melanoma [29–33].

Our laboratories have focused on patient-derived mouse models of pancreatic cancer [3–8].

To effectively use pancreatic cancer patient-derived xenograft (PDX) models in translational research, successful PDX engraftment of surgical specimens in immune-deficient mice is needed. In our previous study, a total of 102 patients underwent pancreatic cancer resection. Tumor tissue from all patents was implanted subcutaneously into mice and tumor engraftment and growth in mice were determined. Multivariate analysis showed that tumor size of more than 3.5 cm in the patient was a significant factor related to successful PDX engraftment. In contrast, there was no correlation of engraftment with surgical procedure, time needed to remove the specimen, tumor differentiation, lymph node metastasis, and protein expression of p53, receptor tyrosine-protein kinase erbB-2 (C-erbB-2), or deleted in pancreatic carcinoma locus 4 (DPC4) [34].

Formalin-fixed, paraffin-embedded (FFPE) specimens from pancreatic cancer patients were used for next generative sequencing (NGS) of DNA. This technology was applied to orthotopic and heterotopic pancreatic cancer models. The expression of genes PEAK1 and MST1R differed by over 100-fold in the two types of models [35].

Our goal in the present study was to further compare the molecular-genetic and metabolic characteristics of PDOX and PDHX models and determine if the characteristics of the original patient tumors were maintained in the models. Toward this goal, PDOX and PDHX models were established simultaneously using the same patient tumor in a series of 25 patients. We compared how similar the two models were to the original patient tumor, and to each other.

## RESULTS

### Establishment and characteristics of PDOX and PDHX mouse models

From January 2015 to December 2015, tumors were collected from 25 pancreatic cancer patients undergoing surgery for pancreatic cancer and used for establishing simultaneous PDHX and PDOX models in NSG mice. The average age of all patients was 61.8 years. Nineteen of the 25 patients (76%) had undergone pancreatico-duodenectomy with a mean tumor size of 3.35 cm. All patients were diagnosed at stage T3, and 20 patients (80%) had lymph node metastasis at diagnosis. Eighteen patients (72%) had p53 inactivation, 7 patients (28%) had receptor tyrosine-protein kinase erbB-2(c-erbB-2) overexpression, and 11 patients (44%) showed deleted in pancreatic carcinoma locus 4(DPC4) inactivation. The two models were established simultaneously using the tumor of a single patient. Tumor formation was observed for at least 6 months after tumor implantation. The established tumors persisted for at least three passages through re-transplantation in other NSG mice. In total, 17 cases of PDOX (68.0%) and 18 cases of PDHX (72.0%) were established, indicating a similar high success rate of each model (Table 1). Thirteen cases (52.0%) were established in both models, and four cases (16.0%) were not established in either model (Supplementary Table 1).

**Table 1:** Host and tumor characteristics for PDX establishment in pancreatic cancer

Characteristics	All (*n* = 25, 100%)	PDX generation (*n*, %)			PDOX & PDHX generation (*n*, %)		
		PDOX (*n* = 17, 68.0%)	PDHX (*n* = 18, 72.0%)	*P* value	both (*n* = 13, 52.0%)	none (*n* = 12, 48.0%)	*P* value
Age, y							
mean (SD)	61.8 ± 8.2	60.23 ± 8.51	62.17 ± 7.69	0.486	60.8 ± 8.07	63.1 ± 8.73	0.498
Gender, *n* (%)							
Female	14 (56%)	11 (31.4%)	12 (34.3%)		9 (36.0%)	5 (20.0%)	
Male	11 (44%)	6 (17.1%)	6 (17.1%)	0.903	4 (16.0%)	7 (28.0%)	0.238
Type of operation, *n* (%)							
PD	19 (76%)	12 (34.3%)	13 (37.1%)		8 (32.0%)	11 (44.0%)	
DP	6 (24%)	5 (14.3%)	5 (14.3%)	0.915	5 (20.0%)	1 (4.0%)	0.160
Tumor size (cm)							
mean (SD)	3.35 ± 0.9	3.48 ± 1.01	3.40 ± 1.02	0.825	3.5 ± 1.10	3.3 ± 0.68	0.589
Tumor differentiation, *n* (%)							
WD	1 (4%)	1 (2.9%)	1 (2.9%)		1 (4.0%)	0 (0%)	
MD	22 (88%)	14 (40.0%)	15 (42.9%)		4 (40.0%)	12 (48.0%)	
PD	2 (8%)	2 (5.7%)	2 (5.7%)	0.997	2 (8.0%)	0 (0%)	0.207
T stage, *n* (%)							
T3	25 (100%)	17 (48.6%)	18 (51.4%)		13 (52.0%)	12 (48.0%)	
N stage, *n* (%)							
N0	5 (20%)	4 (11.4%)	5 (14.3%)		3 (12.0%)	2 (8.0%)	
N1	20 (80%)	13 (37.1%)	13 (37.1%)	0.774	10 (40.0%)	10 (40.0%)	0.689
M stage, *n* (%)							
M0	23 (92%)	15 (42.9%)	16 (45.7%)		11 (44.0%)	12 (48.0%)	
M1^*^	2 (8%)	2 (5.7%)	2 (5.7%)	0.952	2 (8.0%)	0 (0%)	0.157
p53, *n* (%)							
normal	7 (28%)	5 (14.3%)	4 (11.4%)		3 (12.0%)	4 (16.0)	
inactivated	18 (72%)	12 (34.3%)	14 (40.0%)	0.627	10 (40.0%)	8 (32.0%)	0.568
C-erbB-2, *n* (%)							
normal	18 (72%)	14 (40.0%)	14 (40.0%)		10 (40.0%)	8 (32.0%)	
overexpressed	7 (28%)	3 (8.6%)	4 (11.4%)	0.735	3 (12.0%)	4 (16.0%)	0.568
DPC 4, *n* (%)							
normal	14 (56%)	11 (31.4%)	9 (25.7%)		7 (28.0%)	7 (28.0%)	
inactivated	11 (44%)	6 (17.1%)	9 (25.7%)	0.380	6 (24.0%)	5 (20.0%)	0.821

PD, pancreatico-duodenectomy; DP, distal pancreatectomy; WD, well differentiated; MD, moderately differentiated; PD, poorly differentiated; DPC4, deleted in pancreatic carcinoma locus 4. ^*^All of the metastases were liver.

No significant differences (*p* > 0.05) were found in the clinical data of patients with tumors that were successfully established in both models (*n* = 13) compared to the others (*n* = 12) (Table 1). Four of the 13 PDXs established in both models (Nos. 1, 7, 16, and 25) were selected for comparison of homology and heterogeneity between the PDOX/PDHX tumors and the original patient tumor (Supplementary Table 1).

### Histological comparison of the original patient tumors and PDOX and PDHX models

**Figure 1:**
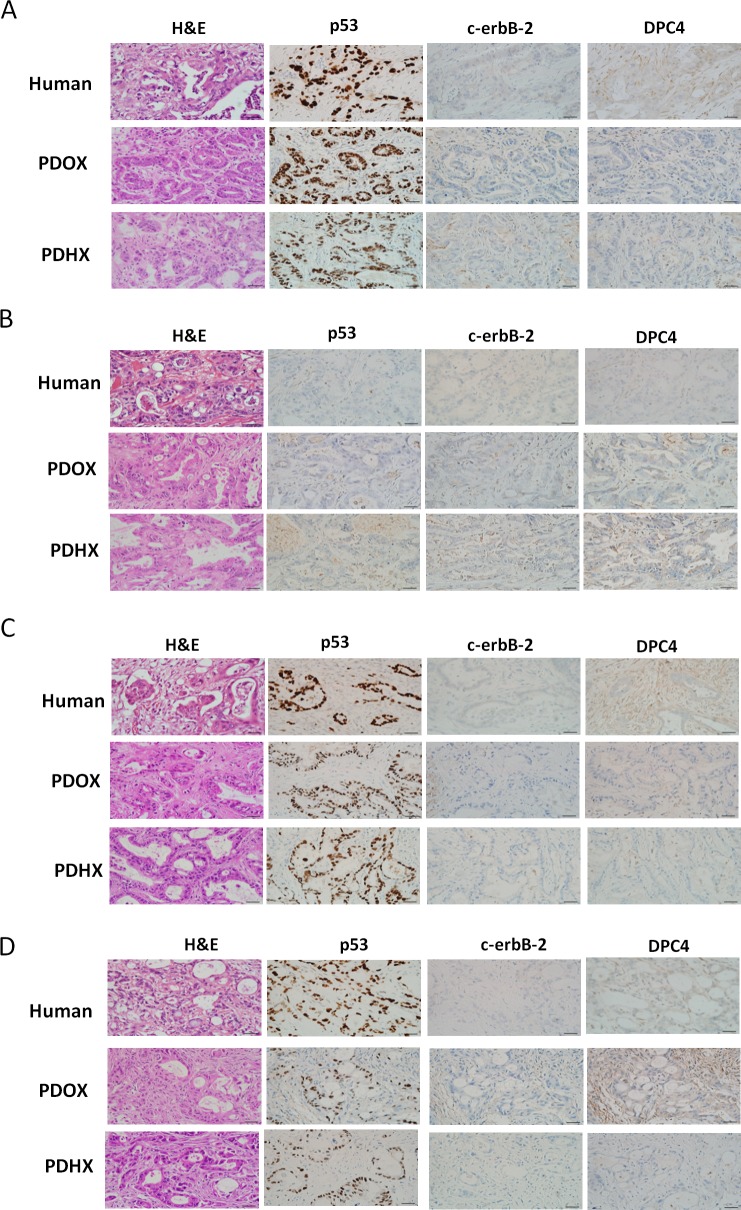
Immunohistochemistry analysis of the original patient tumors compared with patient-derived xenografts PDOX and PDHX Images of H&E, p53, C-erbB-2, and DPC4 staining of patient–PDXs pairs. **A**–**D** refer to patient sets No. 1, 7, 16, and 25, respectively. (Size bar : 20 μm).

Hematoxylin and eosin (H&E) staining was carried out. The pancreatic ducts identified in the H&E staining of the original patient tumor were similar in the corresponding PDOX and PDHX groups (Figure 1).

### Immunohistochemical analysis of gene expression

Immunohistochemistry analysis was performed using anti-P53, -c-erbB-2, and -DPC4 antibodies (Figure 1). As shown in Supplementary Table 1, the degree of expression in each primary carcinoma was quantified, and these findings were confirmed by experienced pancreatic cancer pathologists. A similar degree of expression of p53, c-erbB-2, and DPC4 was detected in the PDOX and PDHX models and the original patient tumor.

### SNP comparison among the original patient tumors and the PDOX and PDHX models

**Figure 2:**
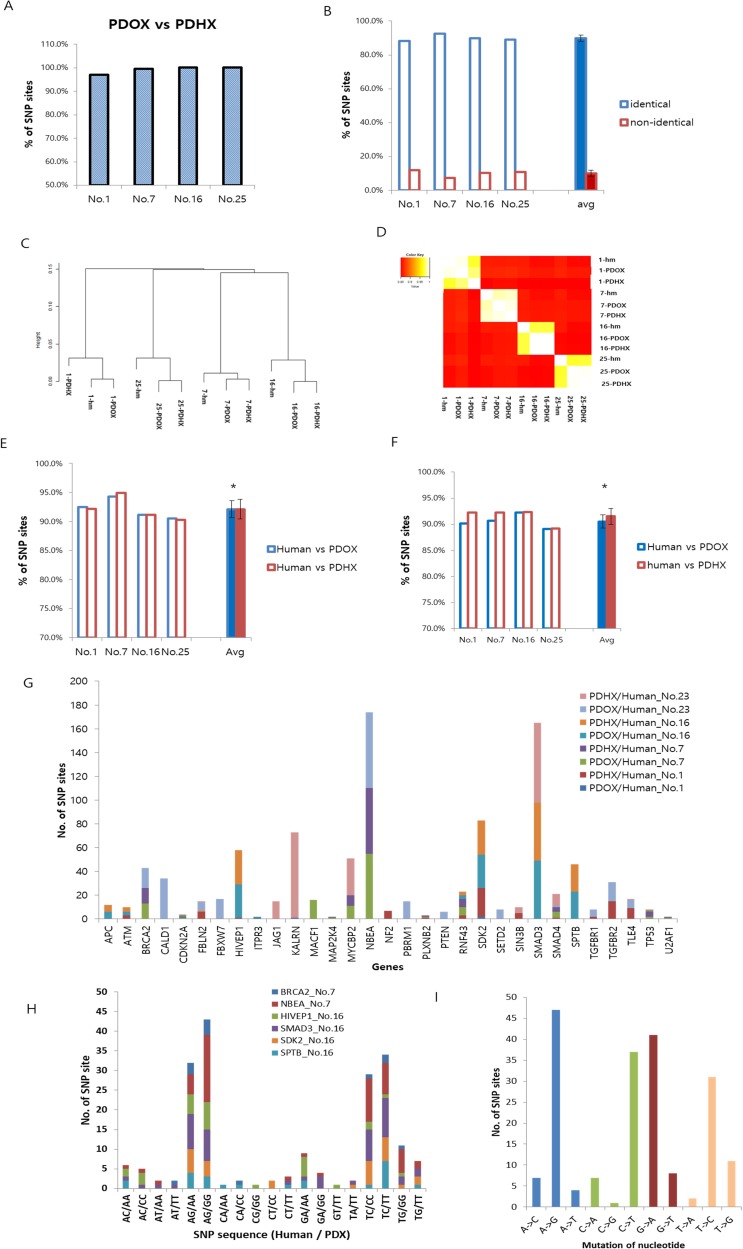
Comparative analysis of single-nucleotide polymorphism (SNP) arrays between the original patient tumor and PDOX and PDHX (**A**) Homology between PDOX and PDHX for cancer-specific SNP sites. At least 96.8% of the SNPs were identical. (**B**) SNPs in original patient tumors were compared to those in both the PDOX and PDHX models. There was a slight difference in each group, but the average identification rate was 89.9%. (**C**) Phylogenetic tree showing group clustering according to the patient tumor. (**D**) Information-based similarity (IBS) matrix based on the SNP variants among human tumors and PDXs. (**E**) Homology between human tumors and PDXs for all SNP sites. There was no significant difference between the groups (^*^p = 0.985). (**F**) Homology between human tumors and PDXs for cancer-specific SNP sites. There was no significant difference between the groups (^*^p = 0.379). (**G**) Comparative analysis of SNP sites of cancer-specific genes. (**H)** Comparison of nucleotide sequences in six representative cases showing a high SNP difference. (**I**) Pattern of nucleotide-sequence changes.

The single nucleotide polymorphism (SNP) profiles of PDOX and PDHX pairs were compared to each other as well as to their original patient tumors. At least 96.8% of the SNPs were found to be identical between the PDOX and PDHX groups (Figure 2A). The SNP patterns among the three groups (original patient cancer, PDOX, and PDHX) showed a similarity rate ranging between 88.2% and 92.7%, with an average of 89.9% of the SNPs of the original patient tumor maintained in both models (Figure 2B). Phylogenetic analysis demonstrated four groups of clusters, corresponding to the respective patient groups (Figure 2C). Information-based similarity-matrix analysis also showed a clear linear correlation, indicating that both xenograft models were most strongly correlated to their respective original tumors.

The original patient tumor and the two PDXs were further analyzed as pairs. For all the SNP sites, the two xenograft models showed an average 92.1% homology to their primary tumors (Figure 2E). For SNP sites of tumor-specific genes, 90.5% of PDOX and 91.5% of PDHX showed the same polymorphisms as the original patient cancer [36] (Figure 2). There was no significant difference from the original patient tumor in either of the two models (*P* > 0.05). To further clarify the differences between each model in cancer-specific gene SNPs, additional analyses were performed for only the 3562 SNP sites that were commonly identified in the analysis of the original patient tumor and PDOX and PDHX models of the four groups (patients no. 1, 7, 16, and 25). The homology of SNPs at these sites was as follows (PDOX vs. PDHX): patient no. 1, 99.9% vs. 97.8%; patient no. 7, 96.9% vs. 97.4%; patient no. 16, 96.0% vs. 95.9%; and patient no. 25, 94.4% vs. 94.3% (Table 2, Figure 2G). As shown in Table 2, the degree of similarity was similar for all genes tested. Six genes [*BRCA2, NBEA* (patient no. 7), *HIVEP1, SMAD3, SDK2,* and *SPTB* (patient no. 16)] with the highest levels of SNPs were then selected for further analysis of SNP patterns. Although the SNP patterns differed from those of the original patient tumor to some extent in the PDX models, they were completely identical between the PDOX and PDHX groups (Supplementary Tables 2 and 3). The most frequently observed mutations were A > G, C > T, G > A, and T > C (Figure 2H, 2I).

**Table 2:** Comparative analysis of the SNP mutations in cancer-related genes of patient-derived xenografts

Gene(s) / No. of SNP sites	Group No.1				Group No.7				Group No.16				Group No.23			
	PDOX/human		PDHX/human		PDOX/human		PDHX/human		PDOX/human		PDHX/human		PDOX/human		PDHX/human	
	O	X	O	X	O	X	O	X	O	X	O	X	O	X	O	X
ACVR2A/39	39	0	39	0	39	0	39	0	39	0	39	0	39	0	39	0
APC/73	73	0	73	0	73	0	73	0	67	6	67	6	73	0	73	0
ARID1A/32	32	0	32	0	32	0	32	0	32	0	32	0	32	0	32	0
ATM/76	76	0	73	3	76	0	76	0	73	3	72	4	76	0	76	0
BCORL1/10	10	0	10	0	10	0	10	0	10	0	10	0	10	0	10	0
BRCA1/35	35	0	35	0	35	0	35	0	35	0	35	0	35	0	35	0
BRCA2/71	71	0	71	0	58	13	58	13	71	0	71	0	54	17	71	0
CALD1/166	166	0	166	0	166	0	166	0	166	0	166	0	132	34	166	0
DISP2/6	6	0	6	0	6	0	6	0	5	1	5	1	6	0	6	0
FBLN2/73	73	0	67	6	73	0	73	0	73	0	72	1	66	7	72	1
FBXW7/87	87	0	87	0	87	0	87	0	87	0	87	0	70	17	87	0
HIVEP1/139	139	0	138	1	139	0	139	0	111	28	110	29	139	0	139	0
ITPR3/91	90	1	91	0	91	0	91	0	90	1	91	0	91	0	91	0
JAG1/49	49	0	49	0	49	0	49	0	49	0	49	0	49	0	34	15
KALRN/539	538	1	539	0	539	0	539	0	539	0	539	0	539	0	467	72
KDM6A/84	84	0	84	0	84	0	84	0	84	0	84	0	84	0	84	0
KRAS/46	46	0	46	0	46	0	46	0	46	0	46	0	46	0	46	0
MACF1/187	187	0	187	0	171	16	187	0	187	0	187	0	187	0	187	0
MAP2K4/3	3	0	3	0	2	1	2	1	3	0	3	0	3	0	3	0
MLL3/122	122	0	122	0	122	0	122	0	122	0	122	0	122	0	122	0
MYCBP2/115	115	0	115	0	104	11	106	9	115	0	115	0	115	0	84	31
NBEA/391	391	0	391	0	336	55	336	55	391	0	391	0	327	64	391	0
NF2/43	43	0	36	7	43	0	43	0	43	0	43	0	43	0	43	0
PBRM1/50	50	0	50	0	50	0	50	0	50	0	50	0	35	15	50	0
PLXNB2/10	10	0	9	1	9	1	9	1	10	0	10	0	10	0	10	0
PTEN/48	48	0	48	0	48	0	48	0	48	0	48	0	42	6	48	0
RBM10/10	10	0	10	0	10	0	10	0	10	0	10	0	10	0	10	0
RNF43/43	43	0	40	3	36	7	36	7	40	3	40	3	43	0	43	0
SDK2/242	240	2	218	24	242	0	242	0	214	28	213	29	242	0	242	0
SETD2/50	50	0	50	0	50	0	50	0	50	0	50	0	42	8	50	0
SF3B1/13	13	0	13	0	13	0	13	0	13	0	13	0	13	0	13	0
SIN3B/20	20	0	15	5	20	0	20	0	20	0	20	0	20	0	15	5
SMAD3/158	158	0	158	0	158	0	158	0	109	49	109	49	158	0	91	67
SMAD4/24	24	0	23	1	19	5	20	4	24	0	24	0	23	1	14	10
SMARCA4/38	38	0	38	0	38	0	38	0	38	0	38	0	38	0	38	0
SPTB/99	99	0	99	0	99	0	99	0	76	23	76	23	99	0	99	0
TGFBR1/27	27	0	25	2	27	0	27	0	27	0	27	0	21	6	27	0
TGFBR2/101	101	0	86	15	101	0	101	0	101	0	101	0	85	16	101	0
TLE4/92	92	0	83	9	92	0	92	0	92	0	92	0	85	7	91	1
TP53/45	45	0	45	0	43	2	41	4	44	1	44	1	45	0	45	0
U2AF1/15	15	0	15	0	15	0	15	0	15	0	15	0	15	0	15	0
Total (%) *N* = 3562	3558 (99.9%)	4 (0.1%)	3485 (97.8%)	77 (2.2%)	3451 (96.9%)	111 (3.1%)	3468 (97.4%)	94 (2.6%)	3419 (96.0%)	143 (4.0%)	3416 (95.9%)	146 (4.1%)	3364 (94.4%)	198 (5.6%)	3360 (94.3%)	202 (5.7%)

**Table 3:** Comparative analysis of DNA methylation in cancer-related genes of patient-derived xenografts

Gene/No. of methylation site	Group No.1				Group No.7				Group No.16				Group No.23			
	PDOX/human		PDHX/human		PDOX/human		PDHX/human		PDOX/human		PDHX/human		PDOX/human		PDHX/human	
	X	O	X	O	X	O	X	O	X	O	X	O	X	O	X	O
		+/−		+/−		+/−		+/−		+/−		+/−		+/−		+/−
ACVR2A/19	17	2/0	19	-	19	-	19	-	19	-	19	-	19	-	19	-
APC/40	40	-	33	1/6	33	1/6	35	0/5	34	0/6	33	0/7	24	10/6	24	12/4
ARID1A/29	29	-	25	2/2	27	2/0	27	2/0	26	2/1	27	1/1	26	3/0	27	2/0
ATM/59	52	7/0	59	-	58	1/0	59	-	59	-	59	-	59	-	59	-
BCORL1/20	19	0/1	20	-	19	0/1	19	0/1	20	-	20	-	20	-	16	3/1
BRCA1/52	51	1/0	45	0/7	36	0/16	37	0/15	48	0/4	45	0/7	51	1/0	50	0/2
BRCA2/19	19	-	18	1/0	18	0/1	18	0/1	19	-	19	-	19	-	19	-
CALD1/57	53	3/0	51	0/6	51	0/6	51	0/6	55	0/2	54	0/3	54	1/2	53	2/2
CDKN2A/7	7	-	7	-	1	6/0	1	6/0	0	7/0	0	7/0	1	6/0	0	7/0
DISP2/18	18	-	16	1/1	17	0/1	17	0/1	16	0/2	16	0/2	16	2/0	16	0/2
FBLN2/50	45	1/4	38	3/9	40	2/8	39	4/7	38	1/11	37	1/12	42	8/0	42	4/4
FBXW7/22	22	-	21	0/1	22	-	22	-	22	-	22	-	20	2/0	21	0/1
HIVEP1/27	27	-	27	-	27	-	27	-	27	-	27	-	25	2/0	26	0/1
ITPR3/50	49	0/1	44	0/6	47	0/3	46	0/4	47	0/3	47	0/3	50	-	48	0/2
JAG1/24	23	1/0	24	-	24	-	24	-	24	-	24	-	23	1/0	23	0/1
KALRN/67	65	1/1	62	0/5	61	0/6	53	0/14	60	0/7	54	0/13	59	8/0	63	0/4
KDM6A/15	15	-	14	0/1	15	-	15	-	14	0/1	14	0/1	14	1/0	14	0/1
KRAS/34	33	0/1	31	0/3	33	0/1	33	0/1	33	0/1	33	0/1	33	1/0	33	0/1
MACF1/87	81	2/4	75	2/10	79	0/8	80	1/6	82	0/5	81	0/6	77	8/2	77	0/10
MAP2K4/25	24	1/0	25	-	25	-	25	-	25	-	25	-	20	4/1	21	4/0
MLL3/24	24	-	22	1/1	22	1/1	24	-	23	1/0	23	0/1	22	2/0	22	2/0
MYCBP2/19	17	2/0	18	1/0	19	-	19	-	19	-	19	-	18	0/1	18	1/0
NBEA/58	57	1/0	51	3/4	48	2/8	48	0/10	51	2/5	53	2/3	53	4/1	52	6/0
NF2/19	19	-	19	-	19	-	19	-	19	-	19	-	19	-	19	-
PBRM1/18	16	2/0	18	-	18	-	18	-	18	-	18	-	18	-	15	2/1
PLXNB2/45	45	-	33	1/11	36	0/9	36	0/9	33	0/12	33	0/12	32	11/2	33	2/10
PTEN/63	63	-	62	0/1	63	-	63	-	61	1/1	61	1/1	61	2/0	61	1/1
RBM10/29	28	0/1	12	0/17	29	-	29	-	29	-	27	2/0	27	2/0	29	-
RNF43/11	10	1/0	8	0/3	7	0/4	7	0/4	7	0/4	7	0/4	8	3/0	8	0/3
SDK2/68	63	0/5	44	5/19	53	5/10	57	3/8	63	0/5	61	0/7	53	12/3	55	6/7
SETD2/24	24	-	24	-	24	-	24	-	24	-	24	-	24	-	24	-
SF3B1/17	17	-	17	-	17	-	17	-	17	-	17	-	17	-	17	-
SIN3B/18	17	0/1	17	0/1	17	0/1	17	0/1	17	0/1	16	0/2	17	1/0	17	0/1
SMAD3/58	58	-	49	0/9	53	0/5	53	0/5	51	0/7	47	0/11	53	5/0	51	3/4
SMAD4/15	11	1/3	14	0/1	14	0/1	14	0/1	15		14	1/0	14	1/0	14	0/1
SMARCA4/44	43	0/1	42	0/2	43	0/1	43	0/1	40	0/4	40	0/4	42	2/0	42	0/2
SPTB/33	32	0/1	30	0/3	29	0/4	31	0/2	30	0/3	30	0/3	30	2/1	30	0/3
TGFBR1/10	8	2/0	9	0/1	9	0/1	9	0/1	9	0/1	9	1/0	8	2/0	8	0/2
TGFBR2/37	36	1/0	32	0/5	34	0/3	34	0/3	33	0/4	33	0/4	31	6/0	33	2/2
TLE4/16	15	0/1	15	0/1	16	-	16	-	16	-	16	-	15	0/1	15	0/1
TP53/38	38	-	37	0/1	38	-	38	-	38	-	37	0/1	38	-	38	-
TP53BP2/20	20	-	20	-	20	-	20	-	20	-	20	-	20	-	20	-
U2AF1/20	20	-	18	1/1	19	1/0	19	1/0	20	-	20	-	18	2/0	19	1/0
Total / 1425	13 70	29/26	12 65	22/138	12 99	21/105	13 02	17/106	13 21	14/90	13 00	16/109	12 90	115/20	12 91	60/74

### DNA methylation analysis comparing the original patient tumors and the PDOX and PDHX models

**Figure 3:**
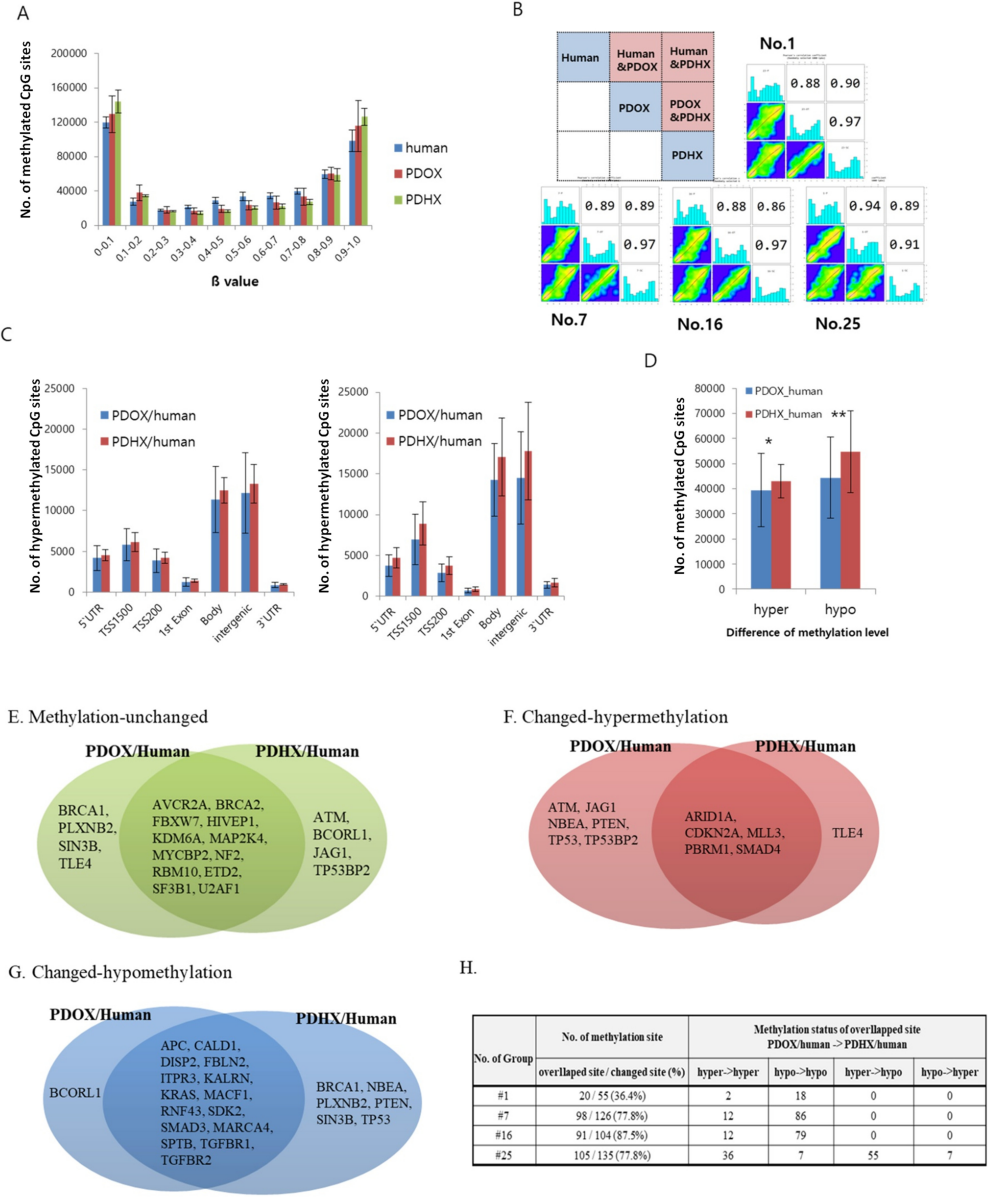
Comparative analysis of DNA methylation arrays between original patient tumors and PDOX and PDHX (**A**) Distribution of methylation levels by group; β values are grouped in 0.1 increments and the percentage of probes is represented for each sample type. (**B**) Pearson's correlation analysis. Of the total methylation sites, 1000 sites were randomly selected. As shown in the box at the top left, the methylation patterns of the original patient tumors, PDOX, and PDHX were compared. The analysis was performed for four human–PDOX and PDHX sets. (**C**) Methylation intensity by genomic compartment. The delta value was calculated by analyzing the methylation-intensity difference between the comparison subjects; hypermethylation was defined as delta ≥ −0.2 and hypomethylation was defined as delta ≤ −0.2. Each methylation level was compared according to the genomic compartment. (**D**) Methylation intensity between two groups. Genomic compartments were not distinguished, and the overall intensity was analyzed together (^*^p = 0.678; ^**^p = 0.401). (**E**–**G**) Comparison of methylation changes for cancer-specific genes: (E) unchanged, (F) hypermethylation, (G) hypomethylation. (**H**) Comparison of methylation patterns for methylation-changed sites.

Genome-wide DNA methylation patterns were analyzed according to methylation intensity among the original patient tumors and PDOX, and PDHX models. For each patient, the 2 PDX models and original-patient tumors showed similar patterns with respect to the number of methylated CpG sites (Figure 3A). In addition, 1000 CpG sites were randomly selected for statistical comparison of methylation homology. As shown in Figure 3B, the original patient tumors showed 89.75% and 88.5% mean similarity to PDOX and PDHX, respectively, among the four cases. PDOX and PDHX showed 95.5% homology. Furthermore, we compared the degrees of hypermethylation and hypomethylation according to genomic compartments in the PDOX and PDHX models relative to the original patient tumors. Although similarity was observed in the methylation patterns of the selected genomic compartments, the PDHX model showed a slight increase in the degree of both hypermethylated and hypomethylated sites (Figure 3C). This difference was evident when analyzing the global hypermethylation and hypomethylation patterns between the PDOX and PDHX models and the original patient tumors; however, there was no statistical significance between the two xenograft models (Figure 3D). Additional analysis of the 1425 methylation sites of cancer-specific genes was performed. As shown in Table 3, a similar number of DNA methylation sites was maintained in the PDOX and PDHX models for each patient (1370 vs. 1265, respectively, in patient no. 1; 1299 vs. 1302, respectively, in patient no. 7;1321 vs. 1300, respectively, in patient no. 16; and 1290 vs. 1291, respectively, in patient no. 25). The average degree of methylation (hyper/hypo) in the four groups was 2.3%/3.6% in the PDOX models and 1.1%/5.5% in the PDHX models (Supplementary Table 4).

Regarding individual gene analysis, the PDOX group had more hypermethylation, whereas the PDHX group had more hypomethylation (Figure 3E–3G). Of the sites with altered methylation patterns, 36.5–87.5% overlapped, and the methylation changes of these overlapping sites were similar in the PDOX and PDHX models (Figure 3H). However, patient no. 25 showed a slightly different pattern from the other groups, similar to the SNP analysis.

### Metabolite analysis comparing the original patient tumors and PDOX and PDHX models

**Figure 4:**
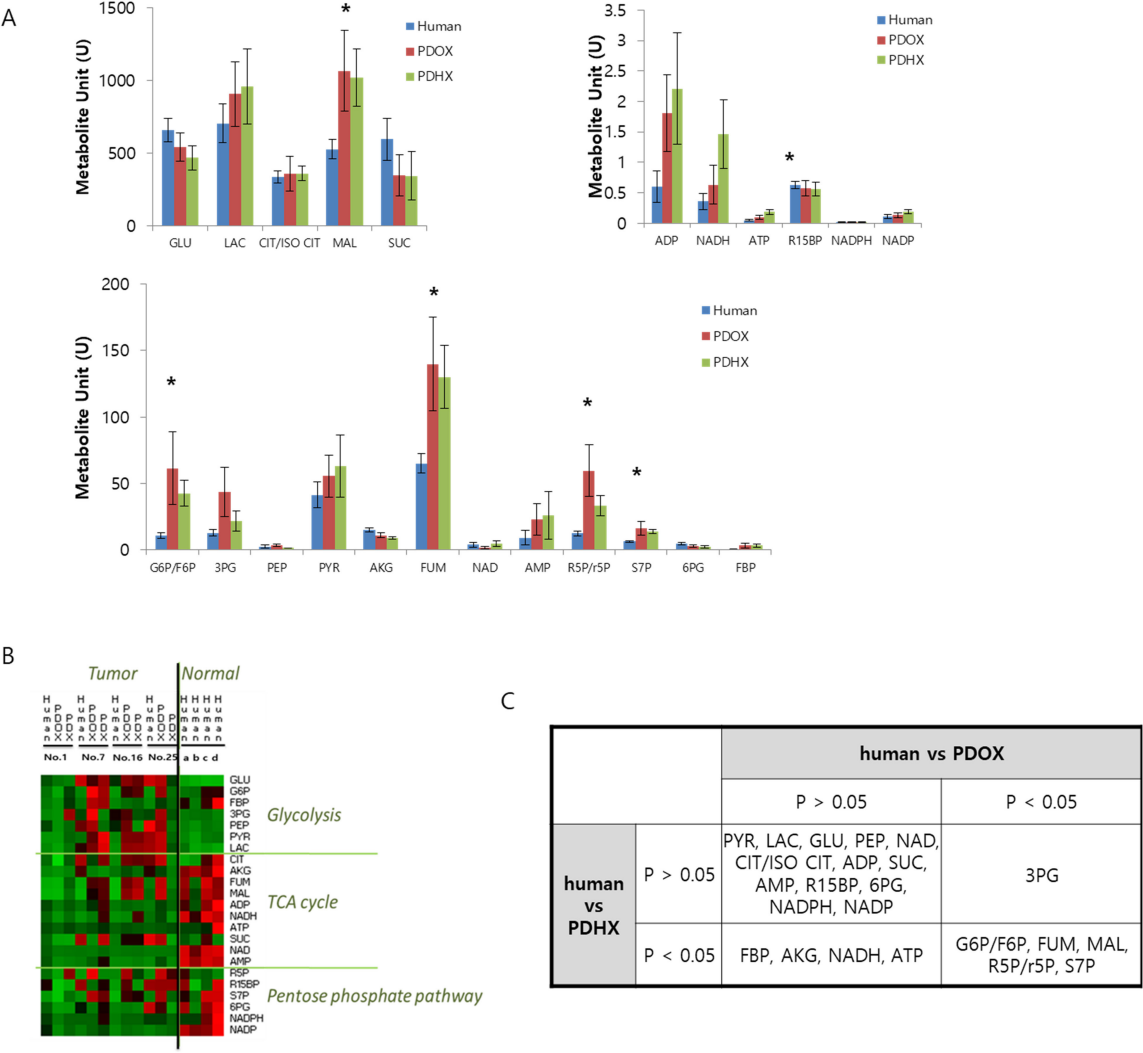
Comparative analysis of metabolite profiling between original patient tumors and PDOX and PDHX tumors (**A**) Quantitative comparison of 23 metabolites in the original patient tumor, PDOX, and PDHX tumor tissues. (^*^p < 0.05) (**B**) Heatmap analysis of metabolites in original patient normal or tumor tissues and PDOX/PDHX tumors. (**C**) Statistical analysis of PDOX and PDHX metabolites, based on the human-metabolite results.

Quantitative comparison of various metabolites was also performed as shown in Figure 4A. Heatmap analysis showed that the original patient tumor tended to have higher amounts of glycolysis metabolites and lower amounts of metabolites related to the tricarboxylic acid (TCA) cycle and pentose-phosphate pathway compared to normal tissues (Figure 4B). When glycolysis metabolites were low in cancer tissues such as the original patient tumor #1 and PDOX and PDHX from this patient, the metabolites of the TCA and pentose-phosphate pathway were also low. By contrast, metabolites related to the TCA cycle and pentose-phosphate pathway were always more highly activated than those related to glycolysis in normal tissues. Accordingly, the tumors that developed in the PDOX and PDX mice generally showed activated glycolysis, known as the Warburg effect. Thirteen of the total 23 metabolites were not significantly different from those of the original primary tumor for both PDOX and PDAX models, and five metabolites differed in both groups (Figure 4C). Specifically, 3-phosphoglyceric acid (3PG) showed a difference from the original patient tumor only in the PDOX model, and metabolites such as fructose 1,6-bisphosphatase (FBP), alpha-ketoglutaric acid (AKG), were found to differ from the original patient tumor only in the PDHX models.

## DISCUSSION

For the simultaneous establishment of PDOX and PDHX models with the same patient tumor, only patients with a relatively large tumor size (average size 3.35 cm) were included in the present study since we previously showed tumors of this size had high PDX-establishment rates [34]. The success rate of the two models was 68% and 72%, respectively, which confirmed that the technology used for PDOX and PDHX establishment is suitable. Of the 25 attempted trials, only four cases did not develop in either model, which was not related to any specific clinical characteristic. To compare the differences between the two models, four of the 13 cases in which both models were successfully established were randomly selected for detailed analysis.

Immunohistochemistry, including H&E staining, showed that the basic phenotype of the original patient tumor was maintained in the PDOX and PDHX models. The molecular-genetic and metabolic pattern was also mostly maintained, as seen in the SNP, DNA methylation microarrays and metabolic analysis.

In this study, the majority of the molecular-genetic characteristics of the original patient tumors are maintained in the PDOX and PDHX models. Although several reports have shown that PDX models can reflect the molecular and genetic characteristics of original patient tumors, the key novelty of the present study is that PDOX and PDHX models were compared to each other and the original patient tumor simultaneously for the first time [34–36]. Many metabolites were maintained in the PDOX and PDHX models with respect to the original patient tumor, confirming the possibility of conducting accurate metabolic analysis of human tumors using PDX models.

The most important point of this study is that major characteristics of gene expression, SNP pattern, DNA methylation, as well as metabolism of the original patient tumors are maintained in the PDOX and PDHX models. It is well known that PDOX models reflect the metastatic pattern of the original patient and PDHX models do not metastasize [1, 2, 16]. A specific tumor microenvironment (TME) may therefore have a great influence on metastasis, rendering the subcutaneous site metastatic resistant [37]. The relationship of DNA hypermethylation and metastasis will be a subject of future experiments.

Previously-developed concepts and strategies of highly-selective tumor targeting can take advantage of molecular targeting of tumors, including tissue-selective therapy which focuses on unique differences between normal and tumor tissues [38–43].

## MATERIALS AND METHODS

### Enrolled patients and clinical information

Twenty-five patients who underwent surgery for pancreatic cancer at Asan Medical Center between February 2015 and June 2016 were included in the study. The surgical specimens were obtained with informed consent from the patients and under permission from the Institutional Review Board (IRB) of Asan Medical Center (No. 2015–0480). A retrospective medical record review was performed to obtain patient-, surgery-, and oncology-related data. The surgical procedure was determined according to the location of the tumor. Either pylorus-preserving or classic pancreatico-duodenectomy was performed to resect tumors of the head or uncinate of the pancreas. Distal pancreatectomy with splenectomy was performed on lesions in the pancreatic body or tail. Oncology-related factors included tumor size, T stage, tumor differentiation, lymphnode metastasis, and genetic alteration of p53, receptor tyrosine-protein kinase erbB-2 (C-erbB-2), or deleted in pancreatic carcinoma locus 4 (DPC4). The surgical specimens and data used in this study were provided by Asan Bio-Resource Center, Korea Biobank Network (2016–3(115)).

### Tumor implantation and passaging in mice

The animal care protocol for this study was approved by the International Animal-Care and Use Committee (IACUC) of the Laboratory of Animal Research at Asan Medical Center, Seoul, Korea. Five-week-old male NOD/SCID (NSG) mice were used for tumor engraftment and were grown in a specific pathogen-free facility. The fresh tumor tissues obtained from pancreatic cancer patients who underwent surgery were immediately placed in RPMI medium (10% FBS, 1% penicillin/streptomycin) at 4°C in a refrigerator. As soon as possible after this, the tumors were mechanically minced into small fragments (1–2 mm^3^). The fragments were implanted subcutaneously in the flank of the mouse for the heterotopic (PDHX) xenograft and on the pancreas for the orthotopic PDOX model [4]. All of the animals were anesthetized with 15 mg/kg Zoletil^®^(Virbac, USA) and 2.5 mg/kg Rompun^®^(Bayer Korea, Korea) by intraperitoneal injection, for tumor implantation. Following implantation, the mice were monitored twice a week for at least 12 months. Once the xenograft tumor had attained a size of 300–500 mm^2^, the tumor was excised and the mice were euthanized following the protocol of the Laboratory of Animal Research at Asan Medical Center. Part of the tumor that had been excised from the mouse was then engrafted into other NSG mice for expansion, while the residual part of the tumor was cryopreserved in a freezing medium with dimethyl sulfoxide and stored in liquid nitrogen.

### Immunohistochemical staining

Tumors were fixed in 10% formalin for at least 24 h and then embedded in paraffin. Both the original patient tumor and mouse-grown tumor tissues were sectioned at 5-μm thickness and stained with hematoxylin and eosin (H&E). Immunohistochemistry was performed to examine the expression levels of p53 and DPC4 in the original patient tumors and in the tumors grown in the PDOX and PDHX models following the protocol of the Department of Diagnostic Pathology at the Asan Medical Center as previously described [44]. In brief, after deparaffinization and antigen retrieval, the slides were labeled with a monoclonal antibody against p53 (clone DO-7, 1:3,000; Dako, Glostrup, Denmark) and DPC4 (clone EP618Y, 1:100; GeneTex, Irvine, CA, USA). Labeling was detected using the avidin-biotin complex staining method. 3,3′-Diaminobenzidine was used as the chromogen for p53, and 3-amino-9-ethylcarbazole was used for detecting DPC4. A pathologist who was experienced in pancreatic cancer reviewed the slides to compare the tumor architecture and desmoplastic appearance in the patient and PDX models.

### Single nucleotide polymorphism (SNP) microarray

An Illumina HumanOmni 2.5M BeadChip, containing 2,500,000 SNPs, was used for whole-genome SNP genotyping. Genomic DNA (200 ng) was denatured with 0.1 N NaOH. Whole-genome amplification was carried out with a random primers mix using a Multi-Sample Master Mix. The amplified DNA was enzymatically fragmented using a Fragmentation Mix followed by precipitation using a Precipitation Mix 1 and 2-propanol. Hybridization of fragmented DNA to BeadChip was performed by denaturing the sample and dispensing 35 μl of the sample onto the BeadChip section followed by incubation for 18 h at 48°C in a hybridization oven. BeadChips were washed, and the staining was performed following single-base extension. This reaction incorporates labeled nucleotides into the extended primers. Genotyping was performed using the Infinium assay on an Illumina GenomeStudio.

### Methylation microarray

The Infinium Human-Methylation450K (HM450K) platform consisting of over 450,000 CpG sites was used to analyze methylation status. In brief, 4 μl of bisulfite-converted DNA (~150 ng) was used in the whole-genome amplification reaction. After amplification, the DNA was fragmented enzymatically, precipitated, and re-suspended in hybridization buffer. All subsequent steps were performed following the standard Infinium protocol. The fragmented DNA was dispensed onto Human Methylation 450 BeadChips and hybridization was performed in a hybridization oven for 20 h. After hybridization, the array was processed through primer extension and an immunohistochemistry-staining protocol to allow for detection of a single-base extension reaction.

### Metabolomics

Standard metabolites and internal standards were purchased from Sigma-Aldrich. All solvents, including water, were purchased from J. T. Baker. The tissue (10–50 mg) was homogenized using a TissueLyzer (Qiagen) with 400 μl chloroform/methanol (2/1). The homogenate was incubated for 20 min at 4°C. Glutamine-13C5, the internal standard, was added to the sample after incubation and mixed well. The sample was then centrifuged at 13000 rpm for 10 min. The supernatant was collected and 100 μl H_2_O was added. The sample was mixed vigorously and centrifuged at 4000 rpm for 20 min, and the upper phase was taken and dried under vacuum. The dried sample was stored at −20°C and reconstituted with 40 μl H_2_O/acetonitrile (50/50 v/v) prior to liquid chromatography-tandem mass spectrometry (LC-MS/MS) analysis. Metabolites were analyzed with an LC-MS/MS system equipped with a 1290 high-performance liquid chromatography (Agilent), Qtrap 5500 (ABSciex), and reverse-phase column (Synergi fusion RP 50 × 2 mm). Three μl of the sample was injected into the LC-MS/MS system and ionized with a turbo spray ionization source. Ammonium acetate (5 mM) in H_2_O and ammonium acetate (5 mM) in acetonitrile were used as mobile phase A and B, respectively. The separation gradient was as follows: hold at 0% B for 5 min; 0–90 % B for 2 min; hold at 90% for 8 min; 90–0% B for 1 min; then hold at 0% B for 9 min. The LC flow rate was 70 μl/min, except between 7–15 min when it was 140 μl/min, and the column temperature was maintained at 23°C. Multiple reaction monitoring was used in negative-ion mode, and the extracted ion chromatogram (EIC) corresponding to the specific transition for each metabolite was used for quantitation. The area under the curve of the EIC was normalized to that of the EIC of the internal standard, and the obtained ratio was used for quantitative comparison.

### Statistical analysis

Statistical analyses were conducted using SPSS for Windows, version 21.0 (IBM Corp., Armonk, NY, USA). The Student's *t*-test or chi-square test was applied depending on the purpose of comparison. *P*-values less than 0.05 were considered statistically significant.

## SUPPLEMENTARY MATERIALS TABLES







## Abbreviations

PDX: patient-derived xenograft
PDOX: patient- derivedorthotopic xenograft
PDHX: patient derived heterotopic xenograft
NSG: NOD scid gamma
SNP: single nucleotide polymorphism
C-erbB-2: receptor tyrosine-protein kinase erbB-2
DPC4: deleted in pancreatic carcinoma locus 4
NAD: nicotinamide adenine dinucleotide
NADP: nicotinamide adenine dinucleotide phosphate
NADH: reduced nicotinamide adenine dinucleotide
NADPH: reduced nicotinamide adenine dinucleotide phosphate
G6P: glucose-6-phosphate
CIT/ISO CIT: citrate / isocitrate
AKG: alpha ketoglutarate
FUM: fumarate
MAL: malate
FBP: Fructose-1; 6-bisphosphate
AMP: adenosine monophosphate
ADP: adenosine diphosphate
ATP: adenosine triphosphate
3PG: 3-phosphoglycerate
6PG: 6-phosphogluconate
PYR: pyruvate
LAC: lactate
GLU: glucose
SUC: succinate
PEP: phosphoenolpyruvate
R5P/r5P: ribulose-5-phosphate /ribose-5-phosphate
R15BP: Ribose-1,5-bisphosphate
S7P: sedoheptulose-7-phosphate
