# Supplementary material for: Genetic and metabolic comparison of orthotopic and heterotopic patient-derived pancreatic-cancer xenografts to the original patient tumors
Source: Oncotarget. 2017 Dec 21;9(8):7867–81. doi: 10.18632/oncotarget.23567 (PMC5814265; doi:10.18632/oncotarget.23567)
Supplement: Supplementary file 1 [file oncotarget-09-7867-s001.pdf]

# Genetic and metabolic comparison of orthotopic and heterotopic patient-derived pancreatic-cancer xenografts to the original patient tumors

## SUPPLEMENTARY MATERIALS

**Supplementary Table 1: Host and tumor characteristics of patient for PDX establishment**

| Case No. | PDOX/PDHX formation | Age/Sex | OP | Tumor size (cm) | Differen tiation | pT/N/ Mstage | p53 | C-erbB2 | DPC4 | Histology |
|---|---|---|---|---|---|---|---|---|---|---|
| 1 | O / O | 57 / F | PD | 2.8 | Mod | 3/1/0 | 3+ | 2+ | 1+ | pancreatic ductal adenocarcinoma |
| 2 | X / O | 59 / M | PD | 2.9 | Mod | 3/1/0 | 3+ | neg | 2+ | pancreatic ductal adenocarcinoma |
| 3 | O / X | 73 / F | PD | 3.7 | Mod | 3/1/0 | neg | neg | neg | pancreatic ductal adenocarcinoma |
| 4 | O / O | 67 / F | PD | 3.5 | Wel | 3/1/0 | 1+ | neg | neg | pancreatic ductal adenocarcinoma |
| 5 | O / O | 60 / F | PD | 2.8 | Mod | 3/1/0 | 3+ | neg | 2+ | pancreatic ductal adenocarcinoma |
| 6 | X / X | 53 / F | DP | 3.7 | Mod | 3/1/0 | 3+ | neg | neg | pancreatic ductal adenocarcinoma |
| 7 | O / O | 53 / F | PD | 3.2 | Mod | 3/1/0 | neg | neg | neg | pancreatic ductal adenocarcinoma |
| 8 | X / O | 68 / M | PD | 2.2 | Mod | 3/1/0 | 3+ | 1+ | 1+ | pancreatic ductal adenocarcinoma |
| 9 | O / O | 50 / M | PD | 2.3 | Mod | 3/0/0 | 3+ | neg | neg | pancreatic ductal adenocarcinoma |
| 10 | X / X | 62 / M | PD | 2.2 | Mod | 3/1/0 | 2+ | 1+ | neg | pancreatic ductal adenocarcinoma |
| 11 | O / X | 50 / M | PD | 3 | Mod | 3/1/0 | 2+ | neg | neg | pancreatic ductal adenocarcinoma |
| 12 | O / O | 60 / M | PD | 4.1 | Por | 3/1/0 | neg | neg | neg | pancreatic ductal adenocarcinoma |
| 13 | X / X | 74 / M | PD | 4 | Mod | 3/1/0 | neg | 2+ | 2+ | pancreatic ductal adenocarcinoma |
| 14 | O / O | 73 / F | DP | 5.4 | Mod | 3/1/0 | 3+ | 1+ | 1+ | pancreatic ductal adenocarcinoma |
| 15 | O / O | 70 / F | PD | 1.7 | Mod | 3/1/0 | 3+ | neg | neg | pancreatic ductal adenocarcinoma |
| 16 | O / O | 58 / F | PD | 3.5 | Mod | 3/1/0 | 3+ | neg | 1+ | pancreatic ductal adenocarcinoma |
| 17 | O / O | 60 / M | DP | 4.9 | Mod | 3/0/1 | 3+ | neg | 1+ | pancreatic ductal adenocarcinoma |
| 18 | X / O | 73 / F | PD | 3.6 | Mod | 3/1/0 | 3+ | neg | 3+ | pancreatic ductal adenocarcinoma |
| 19 | X / O | 68 / F | PD | 3.1 | Mod | 3/0/0 | 2+ | neg | neg | pancreatic ductal adenocarcinoma |
| 20 | O / O | 54 / F | DP | 2.9 | Mod | 3/1/0 | 1+ | 1+ | neg | pancreatic ductal adenocarcinoma |
| 21 | X / X | 66 / M | PD | 3.1 | Mod | 3/1/0 | neg | 1+ | 2+ | pancreatic ductal adenocarcinoma |
| 22 | O / O | 61 / F | PD | 4.5 | Mod | 3/0/0 | neg | neg | neg | pancreatic ductal adenocarcinoma |
| 23 | O / X | 50 / M | PD | 3 | Mod | 3/1/0 | 3+ | neg | neg | pancreatic ductal adenocarcinoma |
| 24 | O / O | 53 / M | DP | 5 | Por | 3/0/1 | neg | neg | neg | pancreatic ductal adenocarcinoma |
| 25 | O / O | 75 / F | DP | 2.8 | Mod | 3/1/0 | 3+ | neg | 3+ | pancreatic ductal adenocarcinoma |

(OP, operation; PD, pancreaticoduodenectomy; DP, distal pancreatectomy; Wel, well differentiated; Mod, moderate differentiated; Por, poor differentiated; DPC4, deleted in pancreatic carcinoma locus 4. * All of the metastases were liver metastasis.

**Supplementary Table 2: SNPs of BRCA2/NBEA among human and PDXs in Group No.7.** See_Supplementary_Table 2

**Supplementary Table 3: SNPs of HIVEP1/SDK2/SMAD3/SPTB among human and PDXs in Group No.16.** See_Supplementary_Table 3

## Supplementary Table 4: Comparative analysis of the DNA methylation of patient derived xenograft in cancer related genes

| Gene | No. of methylation site | PDOX / human | | | PDHX / human | | |
|---|---|---|---|---|---|---|---|
| | | Unchanged | changed | | Unchanged | changed | |
| | | | Hyper-methylation | Hypo-methylation | | Hyper-methylatoin | Hypo-methylation |
| ACVR2A | 19 | 19 | 0 | 0 | 19 | 0 | 0 |
| APC | 40 | 36 | 0 | 4 | 34 | 0 | 6 |
| ARID1A | 29 | 27 | 2 | 0 | 27 | 2 | 0 |
| ATM | 59 | 55 | 4 | 0 | 59 | 0 | 0 |
| BCORL1 | 20 | 19 | 0 | 1 | 20 | 0 | 0 |
| BRCA1 | 52 | 52 | 0 | 0 | 49 | 0 | 3 |
| BRCA2 | 19 | 19 | 0 | 0 | 19 | 0 | 0 |
| CALD1 | 57 | 52 | 0 | 5 | 50 | 0 | 7 |
| CDKN2A | 7 | 1 | 6 | 0 | 4 | 3 | 0 |
| DISP2 | 18 | 17 | 0 | 1 | 16 | 0 | 2 |
| FBLN2 | 50 | 43 | 0 | 7 | 46 | 0 | 4 |
| FBXW7 | 22 | 22 | 0 | 0 | 22 | 0 | 0 |
| HIVEP1 | 27 | 27 | 0 | 0 | 27 | 0 | 0 |
| ITPR3 | 50 | 48 | 0 | 2 | 46 | 0 | 4 |
| JAG1 | 24 | 23 | 1 | 0 | 24 | 0 | 0 |
| KALRN | 67 | 62 | 0 | 5 | 60 | 0 | 7 |
| KDM6A | 15 | 15 | 0 | 0 | 15 | 0 | 0 |
| KRAS | 34 | 33 | 0 | 1 | 33 | 0 | 1 |
| MACF1 | 87 | 78 | 2 | 7 | 77 | 1 | 9 |
| MAP2K4 | 25 | 25 | 0 | 0 | 25 | 0 | 0 |
| MLL3 | 24 | 23 | 1 | 0 | 22 | 2 | 0 |
| MYCBP2 | 19 | 19 | 0 | 0 | 19 | 0 | 0 |
| NBEA | 58 | 57 | 1 | 0 | 57 | 0 | 1 |
| NF2 | 19 | 19 | 0 | 0 | 19 | 0 | 0 |
| PBRM1 | 18 | 15 | 3 | 0 | 17 | 1 | 0 |
| PLXNB2 | 45 | 45 | 0 | 0 | 34 | 0 | 11 |
| PTEN | 63 | 62 | 1 | 0 | 62 | 0 | 1 |
| RBM10 | 29 | 29 | 0 | 0 | 29 | 0 | 0 |
| RNF43 | 11 | 8 | 1 | 2 | 7 | 1 | 3 |
| SDK2 | 68 | 65 | 0 | 3 | 63 | 1 | 4 |
| SETD2 | 24 | 24 | 0 | 0 | 24 | 0 | 0 |
| SF3B1 | 17 | 17 | 0 | 0 | 17 | 0 | 0 |
| SIN3B | 18 | 18 | 0 | 0 | 17 | 0 | 1 |
| SMAD3 | 58 | 53 | 0 | 5 | 53 | 0 | 5 |
| SMAD4 | 15 | 12 | 3 | 0 | 14 | 1 | 0 |
| SMARCA4 | 44 | 43 | 0 | 1 | 42 | 0 | 2 |
| SPTB | 33 | 31 | 0 | 2 | 31 | 0 | 2 |
| TGFBR1 | 10 | 8 | 0 | 2 | 7 | 1 | 2 |
| TGFBR2 | 37 | 33 | 1 | 3 | 33 | 1 | 3 |
| TLE4 | 16 | 16 | 0 | 0 | 15 | 1 | 0 |
| TP53 | 38 | 32 | 6 | 0 | 37 | 0 | 1 |
| TP53BP2 | 20 | 19 | 1 | 0 | 20 | 0 | 0 |
| U2AF1 | 20 | 20 | 0 | 0 | 20 | 0 | 0 |
| Total (%) | 1425 (100%) | 1341 (94.1%) | 33 (2.3%) | 51 (3.6%) | 1331 (93.4%) | 15 (1.1%) | 79 (5.5%) |

( X, unchanged ; O, changed ; +, hypermethylation ; - , hypomethylation )
