# Supplementary material for: Genetic and metabolic comparison of orthotopic and heterotopic patient-derived pancreatic-cancer xenografts to the original patient tumors
Source: Oncotarget. 2017 Dec 21;9(8):7867–81. doi: 10.18632/oncotarget.23567 (PMC5814265; doi:10.18632/oncotarget.23567)
Supplement: Supplementary file 2 [file oncotarget-09-7867-s002.docx]

**Supplementary Table 2. SNPs of BRCA2/NBEA among human and PDXs in Group No.7**

| **IlmnID** | **Genes** | **Chr** | **rs#** | **MapInfo** | **Alleles** | **Ref** | **Human** | **PDOX** | **PDHX** |
| --- | --- | --- | --- | --- | --- | --- | --- | --- | --- |
| kgp10596565-0_T_R_1800304918 | BRCA2 | 13 | rs9534342 | 32955397 | [T/C] | C | TC | CC | CC |
| kgp2554055-0_B_R_1800143143 | BRCA2 | 13 | rs3752451 | 32900933 | [A/T] | T | AT | TT | TT |
| kgp4958940-0_T_F_1800256037 | BRCA2 | 13 | rs11571717 | 32934911 | [A/G] | G | AG | GG | GG |
| kgp595056-0_T_F_1799941877 | BRCA2 | 13 | rs200268825 | 32891452 | [A/G] | A | AG | GG | GG |
| kgp9134715-0_T_F_1800330079 | BRCA2 | 13 | rs1799955 | 32929232 | [A/G] | A | AG | AA | AA |
| rs11571836-131_T_F_1889500066 | BRCA2 | 13 | rs11571836 | 32973439 | [A/G] | A | AG | AA | AA |
| rs144848-131_B_F_1908713595 | BRCA2 | 13 | rs144848 | 32906729 | [A/C] | A | CA | CC | CC |
| rs1799943-131_B_R_1908736944 | BRCA2 | 13 | rs1799943 | 32890572 | [A/G] | G | AG | GG | GG |
| rs206079-131_T_F_1885613746 | BRCA2 | 13 | rs206079 | 32920618 | [A/G] | G | AG | AA | AA |
| rs4942486-131_B_F_1885613751 | BRCA2 | 13 | rs4942486 | 32953388 | [T/C] | T | TC | TT | TT |
| rs517118-131_B_R_1885495066 | BRCA2 | 13 | rs517118 | 32970586 | [A/G] | A | AG | GG | GG |
| rs9534262-131_T_R_1885613750 | BRCA2 | 13 | rs9534262 | 32936646 | [T/C] | T | TC | TT | TT |
| rs9567552-131_B_F_1895009667 | BRCA2 | 13 | rs9567552 | 32890227 | [T/G] | G | TG | GG | GG |
| kgp10498337-0_B_F_1800272905 | NBEA | 13 | rs4943310 | 36222450 | [T/G] | T | TG | GG | GG |
| kgp10632459-0_T_R_1799950978 | NBEA | 13 | rs1461977 | 36085413 | [T/C] | T | TC | CC | CC |
| kgp10826332-0_T_F_1799695442 | NBEA | 13 | rs9574094 | 36103914 | [A/G] | A | AG | AA | AA |
| kgp1370300-0_T_R_1799824741 | NBEA | 13 | rs9544156 | 35950491 | [T/C] | T | TC | CC | CC |
| kgp1543298-0_B_F_1799812266 | NBEA | 13 | rs9565392 | 36159265 | [T/C] | C | TC | CC | CC |
| kgp16552186-0_B_R_1843456709 | NBEA | 13 | rs75114204 | 36067319 | [A/G] | A | AG | GG | GG |
| kgp16606531-0_T_F_1841509870 | NBEA | 13 | rs9574003 | 36053493 | [A/T] | T | AT | AA | AA |
| kgp16608630-0_B_R_1841514069 | NBEA | 13 | rs9635015 | 36125797 | [A/G] | A | AG | GG | GG |
| kgp2282914-0_T_R_1799752489 | NBEA | 13 | rs9565317 | 36049080 | [T/G] | T | TG | TT | TT |
| kgp2478706-0_B_R_1799706441 | NBEA | 13 | rs7332996 | 36036297 | [A/G] | G | AG | GG | GG |
| kgp4894553-0_T_R_1799808125 | NBEA | 13 | rs2874835 | 36022937 | [T/C] | T | TC | CC | CC |
| kgp5602539-0_B_F_1800328551 | NBEA | 13 | rs13329009 | 36155201 | [T/C] | C | TC | CC | CC |
| kgp5784502-0_B_F_1799880028 | NBEA | 13 | rs4943291 | 35545336 | [T/C] | C | TC | TT | TT |
| kgp5889989-0_B_F_1799888440 | NBEA | 13 | rs9530151 | 35652278 | [T/C] | T | TC | TT | TT |
| kgp6821802-0_T_F_1800399939 | NBEA | 13 | rs9544578 | 36110788 | [A/G] | G | AG | GG | GG |
| kgp7275556-0_T_R_1799650011 | NBEA | 13 | rs8002267 | 36111742 | [T/C] | T | TC | TT | TT |
| kgp8157986-0_B_F_1800285455 | NBEA | 13 | rs1461978 | 36085412 | [T/G] | T | TG | GG | GG |
| kgp8332353-0_B_F_1799740258 | NBEA | 13 | rs2798336 | 36171663 | [T/C] | T | TC | CC | CC |
| kgp8986368-0_T_R_1799677709 | NBEA | 13 | rs9543024 | 35603545 | [T/G] | T | TG | GG | GG |
| kgp8995551-0_T_F_1799627206 | NBEA | 13 | rs7324229 | 36238793 | [A/G] | G | AG | GG | GG |
| kgp9114451-0_T_R_1800037510 | NBEA | 13 | rs9565352 | 36089903 | [T/G] | T | TG | GG | GG |
| kgp9689906-0_B_R_1800009514 | NBEA | 13 | rs9573912 | 36009619 | [A/G] | G | AG | GG | GG |
| kgp9852368-0_B_R_1800268876 | NBEA | 13 | rs1031776 | 36047374 | [A/G] | G | AG | GG | GG |
| rs13378709-131_T_R_1890961366 | NBEA | 13 | rs13378709 | 36152425 | [T/G] | T | TG | TT | TT |
| rs1361840-131_T_F_1890939188 | NBEA | 13 | rs1361840 | 36134456 | [A/C] | C | AC | AA | AA |
| rs1381475-131_B_R_1891388438 | NBEA | 13 | rs1381475 | 36072729 | [T/C] | T | CT | CC | CC |
| rs1777668-131_T_R_1890989233 | NBEA | 13 | rs1777668 | 36153301 | [T/C] | T | TC | TT | TT |
| rs2031446-131_T_R_1890996408 | NBEA | 13 | rs2031446 | 36191630 | [A/G] | G | GA | GG | GG |
| rs2050714-131_B_R_1891287276 | NBEA | 13 | rs2050714 | 35898057 | [A/G] | A | AG | GG | GG |
| rs2769332-131_B_R_1891006556 | NBEA | 13 | rs2769332 | 36171537 | [A/G] | A | AG | GG | GG |
| rs2798337-131_B_R_1886407663 | NBEA | 13 | rs2798337 | 36172282 | [A/G] | A | AG | GG | GG |
| rs2798338-131_T_F_1891014384 | NBEA | 13 | rs2798338 | 36172825 | [A/G] | G | AG | AA | AA |
| rs3794387-131_T_R_1967903874 | NBEA | 13 | rs3794387 | 36244990 | [A/G] | A | GA | AA | AA |
| rs4489875-131_B_F_1890926819 | NBEA | 13 | rs4489875 | 36080945 | [T/G] | T | TG | GG | GG |
| rs4606576-131_T_R_1890934243 | NBEA | 13 | rs4606576 | 36122567 | [T/C] | C | TC | TT | TT |
| rs4943303-131_T_R_1908451624 | NBEA | 13 | rs4943303 | 36052726 | [T/C] | T | TC | TT | TT |
| rs589524-131_T_R_1894013701 | NBEA | 13 | rs589524 | 35731964 | [T/C] | T | TC | CC | CC |
| rs7322974-131_T_R_1891007379 | NBEA | 13 | rs7322974 | 36119501 | [T/C] | C | TC | CC | CC |
| rs7332116-131_T_F_1890994959 | NBEA | 13 | rs7332116 | 36078427 | [A/C] | C | AC | CC | CC |
| rs7333310-131_B_F_1890929229 | NBEA | 13 | rs7333310 | 36080957 | [T/C] | T | TC | TT | TT |
| rs7985429-131_T_F_1895035350 | NBEA | 13 | rs7985429 | 36215709 | [A/G] | G | AG | GG | GG |
| rs7999690-131_B_R_1895006867 | NBEA | 13 | rs7999690 | 36109202 | [A/G] | G | AG | AA | AA |
| rs9315349-131_T_F_2093069030 | NBEA | 13 | rs9315349 | 36106282 | [A/G] | A | AG | AA | AA |
| rs9530039-131_T_R_1866903897 | NBEA | 13 | rs9530039 | 35603527 | [T/G] | G | TG | GG | GG |
| rs9530647-131_B_R_1889664228 | NBEA | 13 | rs9530647 | 36080903 | [A/G] | G | AG | GG | GG |
| rs9543006-131_B_F_1895053688 | NBEA | 13 | rs9543006 | 35598402 | [T/C] | T | TC | TT | TT |
| rs9543028-131_B_R_1866905411 | NBEA | 13 | rs9543028 | 35603879 | [A/G] | A | AG | GG | GG |
| rs9544534-131_B_R_1895056643 | NBEA | 13 | rs9544534 | 36094716 | [A/G] | G | AG | GG | GG |
| rs9544868-131_T_F_2093069067 | NBEA | 13 | rs9544868 | 36210239 | [A/G] | G | AG | GG | GG |
| rs9565118-131_B_F_1866907592 | NBEA | 13 | rs9565118 | 35814496 | [T/C] | T | TC | CC | CC |
| rs9573389-131_T_R_1895021379 | NBEA | 13 | rs9573389 | 35786929 | [T/C] | T | TC | CC | CC |
| rs9574120-131_T_F_2093068962 | NBEA | 13 | rs9574120 | 36131948 | [A/G] | G | AG | AA | AA |
| rs9574213-131_B_F_2093068937 | NBEA | 13 | rs9574213 | 36172419 | [T/C] | C | TC | CC | CC |
| rs958793-131_B_R_2093069060 | NBEA | 13 | rs958793 | 36076555 | [T/C] | C | CT | TT | TT |
| rs9635009-131_T_F_1894885080 | NBEA | 13 | rs9635009 | 36009566 | [A/G] | G | AG | GG | GG |
| rs981386-131_T_F_2093069055 | NBEA | 13 | rs981386 | 36089537 | [A/G] | A | AG | GG | GG |
