# Supplementary material for: Genetic and metabolic comparison of orthotopic and heterotopic patient-derived pancreatic-cancer xenografts to the original patient tumors
Source: Oncotarget. 2017 Dec 21;9(8):7867–81. doi: 10.18632/oncotarget.23567 (PMC5814265; doi:10.18632/oncotarget.23567)
Supplement: Supplementary file 3 [file oncotarget-09-7867-s003.docx]

**Supplementary Table 3. SNPs of HIVEP1/SDK2/SMAD3/SPTB among human and PDXs in Group No.16**

| **IlmnID** | **Gene** | **Chr** | **rs#** | **MapInfo** | **Alleles** | **Ref** | **Human** | **PDOX** | **PDHX** |
| --- | --- | --- | --- | --- | --- | --- | --- | --- | --- |
| kgp10021342-0_B_R_1804451850 | HIVEP1 | 6 | rs9349068 | 12048127 | [A/G] | G | AG | GG | GG |
| kgp10081424-0_T_F_1805912907 | HIVEP1 | 6 | rs58503060 | 12023247 | [A/G] | A | AG | GG | GG |
| kgp10291211-0_B_R_1803844127 | HIVEP1 | 6 | rs9369103 | 12129438 | [C/G] | G | CG | GG | GG |
| kgp1442341-0_T_F_1803948582 | HIVEP1 | 6 | rs9462452 | 12088677 | [A/C] | A | AC | CC | CC |
| kgp2137384-0_T_F_1804136872 | HIVEP1 | 6 | rs72826090 | 12113477 | [A/C] | C | AC | CC | CC |
| kgp3108497-0_B_R_1803944627 | HIVEP1 | 6 | rs9369081 | 12095392 | [A/G] | A | AG | AA | AA |
| kgp3745781-0_T_F_1804001954 | HIVEP1 | 6 | rs1126472 | 12125772 | [A/G] | A | AG | AA | AA |
| kgp3776941-0_T_R_1802350932 | HIVEP1 | 6 | rs73724345 | 12062954 | [T/G] | T | TG | TT | TT |
| kgp6928545-0_B_F_1802336693 | HIVEP1 | 6 | rs4339450 | 12085025 | [T/C] | C | TC | TT | TT |
| kgp8166085-0_B_R_1805908257 | HIVEP1 | 6 | rs12210350 | 12048666 | [A/G] | A | AG | GG | GG |
| kgp8413676-0_T_F_1805942157 | HIVEP1 | 6 | rs9349070 | 12051484 | [A/C] | A | AC | AA | AA |
| kgp9907449-0_B_R_1805802357 | HIVEP1 | 6 | rs58363859 | 12066395 | [A/G] | G | AG | GG | GG |
| rs10947756-131_B_R_1895081853 | HIVEP1 | 6 | rs10947756 | 12087892 | [A/G] | G | AG | AA | AA |
| rs12234132-131_B_R_1893991032 | HIVEP1 | 6 | rs12234132 | 12042205 | [A/C] | A | AC | AA | AA |
| rs12525800-131_B_R_1895105930 | HIVEP1 | 6 | rs12525800 | 12035329 | [A/C] | C | AC | CC | CC |
| rs2228220-131_T_F_1967897057 | HIVEP1 | 6 | rs2228220 | 12123249 | [A/G] | A | AG | AA | AA |
| rs2327508-131_T_F_1894001333 | HIVEP1 | 6 | rs2327508 | 12078712 | [A/G] | A | AG | GG | GG |
| rs3734271-131_B_R_1911653136 | HIVEP1 | 6 | rs3734271 | 12129936 | [T/G] | T | GT | TT | TT |
| rs6910367-131_B_F_1891393962 | HIVEP1 | 6 | rs6910367 | 12093331 | [T/C] | T | TC | TT | TT |
| rs6925772-131_T_R_1891309056 | HIVEP1 | 6 | rs6925772 | 12020241 | [T/C] | T | TC | CC | CC |
| rs7741589-131_T_R_1911654836 | HIVEP1 | 6 | rs7741589 | 12129047 | [T/C] | T | TC | CC | CC |
| rs7747677-131_B_R_1895098082 | HIVEP1 | 6 | rs7747677 | 12159112 | [A/G] | A | AG | AA | AA |
| rs9296237-131_T_R_1866881619 | HIVEP1 | 6 | rs9296237 | 12033251 | [T/C] | T | TC | CC | CC |
| rs9296262-131_T_R_1895007709 | HIVEP1 | 6 | rs9296262 | 12092498 | [T/C] | C | TC | CC | CC |
| rs9349054-131_B_R_1866888194 | HIVEP1 | 6 | rs9349054 | 12029312 | [A/G] | G | AG | GG | GG |
| rs9394517-131_T_R_1895122438 | HIVEP1 | 6 | rs9394517 | 12080649 | [T/G] | G | TG | GG | GG |
| rs9394520-131_T_F_1895005672 | HIVEP1 | 6 | rs9394520 | 12083440 | [A/G] | G | AG | GG | GG |
| rs9470903-131_B_F_1866898451 | HIVEP1 | 6 | rs9470903 | 12076171 | [T/C] | T | TC | CC | CC |
| kgp10295493-0_B_F_1810220586 | SDK2 | 17 | rs12450312 | 71514627 | [T/A] | T | TA | AA | AA |
| kgp10656775-0_T_R_1810089641 | SDK2 | 17 | rs7213564 | 71536745 | [T/C] | C | TC | CC | CC |
| kgp11482936-0_B_F_1810199205 | SDK2 | 17 | rs4789148 | 71555674 | [T/C] | T | TC | TT | TT |
| kgp2791604-0_B_R_1810224821 | SDK2 | 17 | rs11655380 | 71535777 | [A/G] | A | AG | AA | AA |
| kgp3654146-0_B_F_1810112483 | SDK2 | 17 | rs790083 | 71577763 | [T/C] | T | TC | TT | TT |
| kgp3765630-0_T_R_1810374660 | SDK2 | 17 | rs7222264 | 71469621 | [T/A] | A | TA | TT | TT |
| kgp4026532-0_T_R_1810002164 | SDK2 | 17 | rs8071109 | 71554010 | [T/C] | C | TC | CC | CC |
| kgp4892253-0_B_R_1810174625 | SDK2 | 17 | rs76940148 | 71548670 | [A/G] | A | AG | GG | GG |
| kgp6175422-0_T_R_1810139666 | SDK2 | 17 | rs35858002 | 71456283 | [T/C] | C | TC | CC | CC |
| kgp6183608-0_B_F_1810551803 | SDK2 | 17 | rs9892036 | 71456279 | [T/G] | G | TG | TT | TT |
| kgp7658180-0_T_F_1810867514 | SDK2 | 17 | rs75114391 | 71535956 | [A/G] | G | AG | GG | GG |
| kgp9184469-0_B_F_1809981221 | SDK2 | 17 | rs34494368 | 71452208 | [T/C] | C | TC | TT | TT |
| kgp9484135-0_B_F_1810858391 | SDK2 | 17 | rs35669214 | 71372662 | [T/C] | T | TC | TT | TT |
| rs12232483-131_B_R_1891371572 | SDK2 | 17 | rs12232483 | 71454537 | [A/G] | G | AG | GG | GG |
| rs12449558-131_B_F_1894920151 | SDK2 | 17 | rs12449558 | 71518773 | [T/C] | T | TC | CC | CC |
| rs1681471-131_T_F_1891284709 | SDK2 | 17 | rs1681471 | 71575062 | [A/G] | A | AG | AA | AA |
| rs1872087-131_T_R_1891284362 | SDK2 | 17 | rs1872087 | 71536725 | [T/G] | T | TG | TT | TT |
| rs4789155-131_T_F_1891301331 | SDK2 | 17 | rs4789155 | 71558419 | [A/G] | G | AG | GG | GG |
| rs4789638-131_T_F_1891339409 | SDK2 | 17 | rs4789638 | 71460143 | [A/G] | G | AG | AA | AA |
| rs4969113-131_B_F_1891302781 | SDK2 | 17 | rs4969113 | 71361834 | [T/C] | T | TC | CC | CC |
| rs6501638-131_T_R_1894014984 | SDK2 | 17 | rs6501638 | 71423391 | [T/C] | T | TC | CC | CC |
| rs6501651-131_B_R_1891411599 | SDK2 | 17 | rs6501651 | 71549061 | [A/G] | A | AG | AA | AA |
| rs7406421-131_B_F_1894809205 | SDK2 | 17 | rs7406421 | 71525426 | [T/G] | G | TG | GG | GG |
| rs790088-131_T_F_1895103437 | SDK2 | 17 | rs790088 | 71588183 | [T/C] | C | CT | CC | CC |
| rs8064680-131_T_F_1891395959 | SDK2 | 17 | rs8064680 | 71514527 | [A/G] | G | AG | AA | AA |
| rs874707-131_T_F_1894925871 | SDK2 | 17 | rs874707 | 71425494 | [T/C] | T | CT | CC | CC |
| rs9890736-131_T_R_1895136824 | SDK2 | 17 | rs9890736 | 71533892 | [T/C] | T | TC | TT | TT |
| rs9893375-131_B_R_1889657785 | SDK2 | 17 | rs9893375 | 71514573 | [A/G] | G | AG | AA | AA |
| rs9913193-131_B_F_1895022645 | SDK2 | 17 | rs9913193 | 71550865 | [T/C] | T | TC | TT | TT |
| kgp10156153-0_B_R_1807286489 | SMAD3 | 15 | rs17213990 | 67394019 | [A/G] | G | AG | AA | AA |
| kgp11000794-0_B_F_1807091753 | SMAD3 | 15 | rs72743422 | 67374222 | [T/C] | C | TC | CC | CC |
| kgp11024907-0_T_R_1806794452 | SMAD3 | 15 | rs11629799 | 67381802 | [T/C] | T | TC | TT | TT |
| kgp11067836-0_B_R_1806741803 | SMAD3 | 15 | rs12904527 | 67471268 | [A/G] | A | AG | GG | GG |
| kgp12410251-0_T_F_1806686553 | SMAD3 | 15 | rs4776896 | 67417089 | [A/T] | T | AT | AA | AA |
| kgp1471800-0_B_R_1807279456 | SMAD3 | 15 | rs9302242 | 67389412 | [A/G] | G | AG | GG | GG |
| kgp205400-0_B_F_1806640609 | SMAD3 | 15 | rs28758234 | 67480480 | [T/C] | C | TC | TT | TT |
| kgp2062317-0_T_F_1807290120 | SMAD3 | 15 | rs55993214 | 67422040 | [A/C] | A | AC | CC | CC |
| kgp227186-0_T_F_1806689107 | SMAD3 | 15 | rs72743427 | 67378734 | [A/G] | A | AG | AA | AA |
| kgp2688400-0_T_F_1806759446 | SMAD3 | 15 | rs12904944 | 67361774 | [A/G] | G | AG | AA | AA |
| kgp2895809-0_B_F_1806931492 | SMAD3 | 15 | rs72743421 | 67371172 | [T/C] | T | TC | TT | TT |
| kgp3244646-0_B_R_1806767089 | SMAD3 | 15 | rs12438366 | 67432515 | [A/G] | A | AG | AA | AA |
| kgp3357591-0_T_F_1807295084 | SMAD3 | 15 | rs62006055 | 67439629 | [A/G] | A | AG | GG | GG |
| kgp3514306-0_T_F_1806783573 | SMAD3 | 15 | rs3784679 | 67469118 | [A/G] | G | AG | GG | GG |
| kgp3559837-0_B_R_1807332056 | SMAD3 | 15 | rs79826987 | 67386084 | [A/G] | G | AG | AA | AA |
| kgp409975-0_B_R_1806864473 | SMAD3 | 15 | rs12901499 | 67370445 | [A/G] | G | AG | AA | AA |
| kgp4104489-0_B_F_1806666105 | SMAD3 | 15 | rs12443188 | 67433941 | [T/A] | T | TA | TT | TT |
| kgp501863-0_B_R_1807277654 | SMAD3 | 15 | rs7181878 | 67389161 | [A/G] | A | AG | AA | AA |
| kgp5233818-0_B_F_1807121163 | SMAD3 | 15 | rs11632964 | 67363573 | [T/C] | C | TC | TT | TT |
| kgp5351844-0_T_R_1806785292 | SMAD3 | 15 | rs10152566 | 67475083 | [T/C] | C | TC | TT | TT |
| kgp5501089-0_B_F_1807352881 | SMAD3 | 15 | rs11631254 | 67417800 | [T/C] | T | TC | TT | TT |
| kgp5550810-0_T_F_1807020280 | SMAD3 | 15 | rs11071934 | 67401977 | [A/G] | G | AG | GG | GG |
| kgp5840279-0_B_R_1807254946 | SMAD3 | 15 | rs62006053 | 67427590 | [A/G] | A | AG | GG | GG |
| kgp6513315-0_T_F_1807339013 | SMAD3 | 15 | rs7178291 | 67419682 | [A/G] | A | AG | GG | GG |
| kgp6521251-0_T_R_1806858177 | SMAD3 | 15 | rs76302777 | 67360226 | [T/C] | C | TC | TT | TT |
| kgp7558846-0_T_F_1807012187 | SMAD3 | 15 | rs9972423 | 67391805 | [A/T] | T | AT | TT | TT |
| kgp7958595-0_B_F_1807272125 | SMAD3 | 15 | rs4776341 | 67414911 | [T/G] | T | TG | TT | TT |
| kgp8700357-0_T_F_1806732500 | SMAD3 | 15 | rs61414048 | 67371929 | [A/G] | G | AG | AA | AA |
| kgp9362301-0_T_R_1807177250 | SMAD3 | 15 | rs56324967 | 67402824 | [T/C] | T | TC | TT | TT |
| kgp9513882-0_B_F_1806983732 | SMAD3 | 15 | rs12439500 | 67421320 | [T/C] | C | TC | CC | CC |
| kgp9811460-0_T_R_1807255054 | SMAD3 | 15 | rs60428975 | 67408487 | [T/C] | T | TC | CC | CC |
| kgp9847533-0_T_R_1807188034 | SMAD3 | 15 | rs62006018 | 67402528 | [T/C] | C | TC | CC | CC |
| rs10518705-131_B_F_1885627365 | SMAD3 | 15 | rs10518705 | 67363283 | [T/G] | T | TG | GG | GG |
| rs10518707-131_B_R_1893988420 | SMAD3 | 15 | rs10518707 | 67365622 | [A/G] | A | AG | GG | GG |
| rs11071938-131_B_F_1891319399 | SMAD3 | 15 | rs11071938 | 67419987 | [T/C] | C | TC | CC | CC |
| rs12900401-131_T_R_1893183549 | SMAD3 | 15 | rs12900401 | 67486590 | [T/C] | C | TC | TT | TT |
| rs12915039-131_B_R_1893183553 | SMAD3 | 15 | rs12915039 | 67434348 | [A/C] | A | AC | AA | AA |
| rs1438386-131_T_R_1885627426 | SMAD3 | 15 | rs1438386 | 67407899 | [A/G] | G | GA | GG | GG |
| rs17293443-131_B_F_1885627464 | SMAD3 | 15 | rs17293443 | 67437863 | [T/C] | T | TC | CC | CC |
| rs2053294-131_T_R_1908325062 | SMAD3 | 15 | rs2053294 | 67399084 | [A/G] | G | GA | AA | AA |
| rs2118610-131_T_F_1885627446 | SMAD3 | 15 | rs2118610 | 67428334 | [T/C] | T | CT | TT | TT |
| rs4776339-131_T_R_1894856459 | SMAD3 | 15 | rs4776339 | 67384150 | [T/C] | C | TC | CC | CC |
| rs4776881-131_B_F_1891381219 | SMAD3 | 15 | rs4776881 | 67376000 | [T/C] | C | TC | CC | CC |
| rs4776890-131_T_R_1885627407 | SMAD3 | 15 | rs4776890 | 67393045 | [T/G] | G | TG | GG | GG |
| rs6494633-131_B_F_1885627441 | SMAD3 | 15 | rs6494633 | 67423306 | [T/C] | C | TC | TT | TT |
| rs7162912-131_B_F_1885502377 | SMAD3 | 15 | rs7162912 | 67361308 | [T/G] | T | TG | TT | TT |
| rs7163381-131_T_F_1885627430 | SMAD3 | 15 | rs7163381 | 67414055 | [A/G] | A | AG | AA | AA |
| rs745103-131_T_R_1885627461 | SMAD3 | 15 | rs745103 | 67435075 | [A/G] | G | GA | GG | GG |
| rs920293-131_T_R_1866877878 | SMAD3 | 15 | rs920293 | 67414425 | [A/G] | A | GA | GG | GG |
| kgp10610778-0_B_R_1807588475 | SPTB | 14 | rs3819932 | 65246227 | [A/C] | C | AC | AA | AA |
| kgp10872070-0_T_F_1807461859 | SPTB | 14 | rs229596 | 65237231 | [A/G] | A | AG | AA | AA |
| kgp11674486-0_B_F_1807586510 | SPTB | 14 | rs170681 | 65236992 | [T/C] | C | TC | CC | CC |
| kgp12315296-0_T_F_1821899353 | SPTB | 14 | rs2269297 | 65274225 | [A/G] | G | AG | AA | AA |
| kgp19703839-0_B_R_1833051633 | SPTB | 14 | rs200224302 | 65254007 | [A/G] | A | AG | GG | GG |
| kgp4275073-0_B_F_1821538494 | SPTB | 14 | rs74056022 | 65269774 | [T/C] | C | TC | TT | TT |
| kgp4478270-0_T_R_1822017134 | SPTB | 14 | rs8017385 | 65280747 | [T/G] | G | TG | TT | TT |
| kgp6877755-0_B_F_1821626562 | SPTB | 14 | rs17767662 | 65280824 | [T/C] | C | TC | TT | TT |
| kgp7304089-0_B_R_1822040702 | SPTB | 14 | rs11851199 | 65256188 | [A/G] | A | AG | GG | GG |
| kgp9022364-0_B_R_1807370716 | SPTB | 14 | rs2285004 | 65272859 | [A/G] | G | AG | AA | AA |
| rs17102161-131_T_F_1893189486 | SPTB | 14 | rs17102161 | 65283958 | [A/G] | G | AG | AA | AA |
| rs1741464-131_T_R_1891409771 | SPTB | 14 | rs1741464 | 65280076 | [A/C] | C | CA | AA | AA |
| rs1741488-131_T_F_1891342326 | SPTB | 14 | rs1741488 | 65245956 | [T/C] | C | CT | TT | TT |
| rs17826105-131_T_R_1891329066 | SPTB | 14 | rs17826105 | 65250828 | [T/C] | C | TC | TT | TT |
| rs17826201-131_B_R_1895068356 | SPTB | 14 | rs17826201 | 65282953 | [A/C] | C | AC | AA | AA |
| rs2277503-131_B_F_1894923863 | SPTB | 14 | rs2277503 | 65271780 | [A/G] | G | GA | AA | AA |
| rs229586-131_T_R_1893189467 | SPTB | 14 | rs229586 | 65263347 | [T/C] | C | TC | TT | TT |
| rs229587-131_B_F_2085978516 | SPTB | 14 | rs229587 | 65263300 | [T/C] | C | TC | TT | TT |
| rs229592-131_T_R_1889650232 | SPTB | 14 | rs229592 | 65241228 | [A/G] | A | GA | AA | AA |
| rs229594-131_T_R_1894001345 | SPTB | 14 | rs229594 | 65238141 | [T/C] | T | TC | TT | TT |
| rs229670-131_T_R_1893189490 | SPTB | 14 | rs229670 | 65283501 | [A/C] | A | CA | CC | CC |
| rs4902317-131_B_R_1891409947 | SPTB | 14 | rs4902317 | 65270024 | [A/G] | A | AG | GG | GG |
| rs6573568-131_T_R_1885620278 | SPTB | 14 | rs6573568 | 65249815 | [T/C] | C | TC | TT | TT |
